# Perspectives of general practitioners and nursing staff on acute hospital transfers of nursing home residents in Germany: results of two cross-sectional studies

**DOI:** 10.1186/s12875-020-01108-x

**Published:** 2020-02-11

**Authors:** Alexander Maximilian Fassmer, Alexandra Pulst, Ove Spreckelsen, Falk Hoffmann

**Affiliations:** 1 Division of Outpatient Care and Pharmacoepidemiology, Department of Health Services Research, School VI - Medicine and Health Sciences, Carl von Ossietzky University of Oldenburg, Oldenburg, Germany; 2 Department of Health Services Research, Institute for Public Health and Nursing Research, University of Bremen, Bremen, Germany; 3 Health Sciences, University of Bremen, Bremen, Germany; 4 Division of General Practice, Department of Health Services Research, School VI - Medicine and Health Sciences, Carl von Ossietzky University of Oldenburg, Oldenburg, Germany

**Keywords:** Nursing home, Nursing home residents, Hospital transfer, Hospitalisation, Emergency department, General practitioner, Nursing staff, Survey

## Abstract

**Background:**

Visits in emergency departments and hospital admissions are common among nursing home (NH) residents and they are associated with significant complications. Many of these transfers are considered inappropriate. This study aimed to compare the perceptions of general practitioners (GPs) and NH staff on hospital transfers among residents and to illustrate measures for improvement.

**Methods:**

Two cross-sectional studies were conducted as surveys among 1121 GPs in the German federal states Bremen and Lower Saxony and staff from 1069 NHs (preferably nursing staff managers) from all over Germany, each randomly selected. Questionnaires were sent in August 2018 and January 2019, respectively. The answers were compared between GPs and NH staff using descriptive statistics, Mann-Whitney U tests and χ2-tests.

**Results:**

We received 375 GP questionnaires (response: 34%) and 486 NH questionnaires (response: 45%). GPs estimated the proportion of inappropriate transfers higher than NH staff (hospital admissions: 35.0% vs. 25.6%, *p* < 0.0001; emergency department visits: 39.9% vs. 20.9%, p < 0.0001). The majority of NH staff and nearly half of the GPs agreed that NH residents do often not benefit from hospital admissions (NHs: 61.4% vs. GPs: 48.8%; *p* = 0.0009). Both groups rated almost all potential measures for improvement differently (*p* < 0.0001), however, GPs and NH staff considered most areas to reduce hospital transfers importantly. The two most important measures for GPs were more nursing staff (91.6%) and better communication between nursing staff and GP (90.9%). NH staff considered better care / availability of GP (82.8%) and medical specialists (81.3%) as most important. Both groups rated similarly the importance of explicit advance directives (GPs: 77.2%, NHs: 72.4%; *p* = 0.1492).

**Conclusions:**

A substantial proportion of hospital transfers from NHs were considered inappropriate. Partly, the ratings of possible areas for improvement differed between GPs and NH staff indicating that both groups seem to pass the responsibility to each other. These findings, however, support the need for interprofessional collaboration.

## Background

In the upcoming decades, especially western countries will continue to age, leading to a further increase of care-dependent persons [1–3]. Accompanying that development, the proportion of older persons living in nursing homes (NHs) will keep on growing. In Germany, nearly 800,000 people live in NHs [4]. Due to increased frailty and vulnerability [5–7] residents are at higher risk for (acute) hospital transfers than the older community-dwelling population [8–11]. These visits in emergency departments (EDs) with subsequent discharge to the NH (in the following named ED visits) and hospital admissions are even more common in Germany than in other Western countries [12, 13] with 0.5 ED visits and 1.2 hospital admissions per resident and year [14]. Furthermore, a comparably high proportion (30%) of German NH residents die in hospital [15, 16].

The major reasons for acute hospital transfers of NH residents are falls and injuries, cardiovascular diseases, respiratory diseases and infections [8, 10, 13, 17, 18]. In general, hospital transfers of older persons are associated with significant complications, e.g. further functional and mental decline or nosocomial infections [19, 20], and lead to a high use of healthcare resources [19, 21]. Thus, the existing evidence considers many of these transfers inappropriate or potentially avoidable [21–24]. However, there are numerous instruments judging the appropriateness of hospital transfers [24, 25], and the according proportions of inappropriate transfers vary substantially between 2 and 77% [26, 27]. In many cases solely specific medical diagnoses belonging to the so called ambulatory care sensitive conditions (ACSCs) were used, for instance, heart failure and pneumonia [18, 28–32]. However, most of these proxies do not consider the heterogeneity of situations and the extent of factors influencing the decision for hospital transfers of NH residents [21, 24, 33–36].

Due to the complexity of the decision for hospital transfers [37], inquiring and understanding the perceptions of healthcare professionals directly involved in this process seems to be a more appropriate way aiming to reduce unnecessary transfers [33, 35, 36, 38–43]. Usually, nursing staff are the first ones identifying a resident’s deterioration and they know the complexity of the decision-making process [37]. General practitioners (GPs) provide most medical care for NH residents [4, 44] and both groups play essential roles for the transfer decision [36, 41, 43, 45, 46]. In Germany, NH residents can be admitted to a hospital with a physician’s (e.g., GP’s) referral, however, the transfer process can also be initiated by the NH staff calling the emergency medical service without involvement of a physician which is common practice in German speaking countries [23, 47]. Several approaches discussed for reducing inappropriate hospital transfers include better interprofessional collaboration [25, 33, 37, 41, 48]. However, most of the existing literature on reasons for hospital transfers of NH residents and areas for improvement only focussed on just one of these healthcare professionals [35, 37, 40, 43, 48], or had small sample sizes [39, 49, 50]. Moreover, research on this topic from Germany is rare.

To sum up, NH staff and GPs are the main groups for estimating the (medical) care needs of residents and their views on hospital transfers are essential in investigating appropriateness and measures for improvement. Therefore, this explorative study aims to assess and compare GPs’ and nursing staff’s perspectives on hospital transfers from NHs.

## Methods

### Study design

For these cross-sectional studies we surveyed GPs in two German federal states and NH staff from all over Germany. Both studies were part of the HOMERN project (HOspitalisations and eMERgency department visits of Nursing home residents), funded by the Innovation Fund coordinated by the Innovation Committee of the Federal Joint Committee (G-BA) in Germany. The project explores, in depth, health care of NH residents with a focus on hospital transfers.

For the GP survey the sample size calculation was based on a UK survey among multidisciplinary healthcare professionals (including physicians and nurses) with direct experience in acute care of NH residents. The respondents considered 55% of hospital admissions inappropriate [39]. For estimating a 95% confidence interval (CI) with a precision of ±5% (50–60%) (calculation performed with OpenEpi Version 3.01) we needed a sample size of 381 GPs. Assuming a response of 34% as in a previous survey among German GPs [51] a gross sample of 1121 respondents was necessary. This number was randomly selected from all registered GPs (including general internists working in primary care; approx. *n* = 5500) listed by the Associations of the Statutory Health Insurance Physicians (“Kassenärztliche Vereinigungen”) in the federal states of Bremen and Lower Saxony. We used the same sample size of originally 1121 facilities for the survey among NH staff. Basic data of these NHs (name, address) were also randomly drawn from all approx. 11,200 NHs providing long-term care in Germany listed in the Care Navigator provided by the Federal Association of Local Health Insurance Funds (“AOK Pflege-Navigator”). After checking the sample manually for inclusion criteria, we excluded 52 facilities as they were no longer in place or were caring mostly for children, patients in persistent vegetative state or with prolonged mechanical ventilation, resulting in a final sample size of 1069 NHs.

Both surveys followed an identical methodological approach. We used a number of strategies found to be effective to increase response to postal questionnaires by a Cochrane review [52], including a short questionnaire, follow-up contact, providing a second copy of the questionnaire at follow-up, personalized letters and university origin. The GP data already contained the physicians’ names to which we addressed the questionnaire. Since for the NH survey the letters were preferably addressed to the nursing staff manager, we searched their names manually. If the respective nursing staff manager’s name could not be found, we used the name of the NH director or the executive board instead, if available. Only if no contact person was detectable the questionnaire was addressed to the current nursing staff manager in the respective facility.

In August 2018, we invited the GPs by postal letter with a paper-based questionnaire and sent all of them a reminder letter (with a second copy of the questionnaire attached) after two weeks. The same approach was used for the NHs in January 2019. Data in both surveys were collected anonymously.

### Content of the questionnaire

The four-page questionnaires on medical care in NHs, hospital transfers (including ED visits and hospital admissions), and end-of-life care of NH residents was developed by a multidisciplinary research team of health scientists and GPs. It was pretested with non-involved GPs, whose comments were incorporated into the final version. The current article covers the issues regarding hospital transfers for which the same questions were used for GPs and NH staff. This original version of the questionnaire on GPs can be found in the Additional file 1, the original questionnaire in NH staff can be found in Strautmann et al. [53].

First, we asked the participants to estimate the proportion of inappropriate hospital admissions and ED visits among NH residents with the question “Taken as a whole, what is the proportion of inpatient hospital stays and outpatient emergency department visits of NH residents you estimate as inappropriate?” (see Additional file 1, question no. 4). Second, we framed four statements containing current courses of action and potential difficulties concerning hospital transfers (see Additional file 1, question no. 5): (1) “Residents often do not benefit from inpatient hospital stays”; (2) “Nursing staff calls too often the emergency medical service without prior medical consultation”; (3) “After falls of NH residents there is often no alternative than a transfer to hospital”; (4) “Hospital transfer decisions should be taken more cautiously for residents with advanced dementia”. The healthcare professionals should assess these on a 5-point Likert scale ranging from ‘0 = totally disagree’ to ‘4 = totally agree’. The third part dealt with possible areas for reducing the number of hospital transfers which the GPs and NH staff should again rate using a 5-point Likert scale ranging from ‘0 = no relevance’ to ‘4 = high relevance’ (see Additional file 1, question no. 6). Drawing from the existing literature [27, 33, 34, 46, 54] and insights from interviews with nurses and GPs in the scope of the HOMERN project we listed the following eight measures: (1) better communication between nursing staff, (2) better communication between nursing staff and GP, (3) better GP’s care/availability, (4) better medical specialist’s care/availability (5) better availability of (medical) resources in the NH (e.g., catheters, rapid diagnostic tests, drugs), (6) more nursing staff, (7) qualification activities for nursing staff, and (8) explicit advance directives (ADs). Besides, the respondents were given the opportunity stating a measure not mentioned before (free-text).

Moreover, the GPs and the NH staff were asked for the following characteristics (see Additional file 1, questions no. 11 and 12): age, sex, location of the medical practice or the NH, respectively (≤2000, ≤5000, ≤20,000, ≤50,000, ≤100,000, more than 100,000 inhabitants), and number of years working as a GP or in the current position in the NH (nursing management, facility administration, executive board, other), respectively. Furthermore, the GPs were requested for number of residents they care for and the NH staff should additionally report the number of beds in the facility and the distance to the nearest hospital with ED.

### Statistical analyses

Exploratory analyses were conducted to compare responses between GPs and NH staff. We used descriptive statistics and calculated frequencies for categorical data presenting as n (%). For continuous data we stated the mean with standard deviation (SD) and the range. The assessed proportions of inappropriate hospital transfers were compared between GPs and NH staff by Mann-Whitney *U* test. Responses regarding statements containing current courses of action and potential deficits concerning hospital transfers as well as the assessment of possible areas for improvement were compared between both groups using chi-square tests (χ^2^-Test). We combined the items ‘totally disagree’ and ‘disagree’ as well as ‘totally agree’ and ‘agree’ to one item, respectively. The same applies to the items ‘no relevance’ and ‘minor relevance’ as well as ‘major relevance’ and ‘high relevance’. Since not all respondents answered every question in the questionnaire the analyses were restricted to subjects with no missing values given in the respective questions (presented as n in Table 1 + 2). All statistics were calculated using the SAS programme for Windows, version 9.4 (SAS Institute Inc., Cary, North Carolina, United States).

**Table 1:** Characteristics of the respondents

	General Practitioners (*N* = 375)		Nursing Home Staff (*N* = 486)	
Age [years]	(*n* = 371)*		(*n* = 465)*	
Mean (SD)	54.4	(9.3)	48.0	(9.8)
≤ 49	106	(28.6%)	234	(50.3%)
50–59	150	(40.4%)	170	(36.6%)
≥ 60	115	(31.0%)	61	(13.1%)
Sex	(*n* = 373)*		(*n* = 476)*	
Male	215	(57.6%)	138	(29.0%)
Female	158	(42.4%)	338	(71.0%)
Location of the medical practice / the nursing home	(n = 373)*		(*n* = 461)*	
Rural (≤ 20,000 inhabitants)	195	(52.3%)	238	(51.6%)
Semi-urban (> 20,000–≤100,000 inhabitants)	94	(25.2%)	131	(28.4%)
Urban (> 100,000 inhabitants)	84	(22.5%)	92	(20.0%)
Years as general practitioner / in the current position in the nursing home	(n = 373)*		(*n* = 474)*	
Mean (SD)	18.0	(10.8)	9.7	(8.0)
≤ 9	94	(25.2%)	269	(56.8%)
10–19	106	(28.4%)	139	(29.3%)
≥ 20	173	(46.4%)	66	(13.9%)

*numbers differ because of missing values

Since data in both surveys were collected anonymously consent to participate was not required. For both cross-sectional studies, we received waivers from the medical ethics committee of the Carl von Ossietzky University of Oldenburg in Germany (2018–080 and 2018–147).

## Results

### Characteristics of the respondents

Of the 1121 GPs surveyed, 375 returned the questionnaire (response: 33.5%). Most responding physicians worked in group practices or medical care centres (67.0%) and the bulk of respondents worked in rural areas (52.3%). A higher proportion was male (57.6%) and the mean age was 54.4 years (SD: 9.3; range: 33–84). On an average, the physicians cared for 46.8 NH residents (SD: 43.5; range: 0–360) and they had been working as a GP for 18.0 years (SD: 10.8; range 1–48) (see Table 1).

From the target population of 1069 NHs, we received 486 questionnaires (response: 45.5%). Over half of the facilities (52.7%) were non-profit owned, 39.2% were in a private for-profit ownership, and the remaining 8.1% were owned by the respective local community. The majority of the facilities were located in rural areas (51.6%) and the mean distance to the nearest hospital with ED was 8.5 km (SD: 7.8; range: 0–50). On average, 89.1 residents lived in the facilities (SD: 47.5; range: 4–403). The NH staff was younger than the GPs (mean age: 48.0 years; SD: 9.8; range: 27–69) and the proportion of females was substantially higher (71.0%). Most of these respondents were nursing staff managers (64.7%) or NH directors (29.9%) and they had been working in the respective positions for averaging 9.7 years (SD: 8.0; range 0.5–50) (see Table 1).

### Hospital admissions and emergency department visits

The responding GPs and NH staff estimated the proportion of inappropriate hospital transfers differently. On average, the GPs rated 35.0% (SD: 21.9%) of hospital admissions as inappropriate, while 25.6% (SD: 21.5%) of the NH staff made this assessment (*p* < 0.0001). In the same way, the GPs considered ED visits more frequently inappropriate (mean: 39.9%, SD: 24.1%) than the nursing staff (mean: 20.9%, SD: 21.0%; p < 0.0001).

**Fig. 1:**
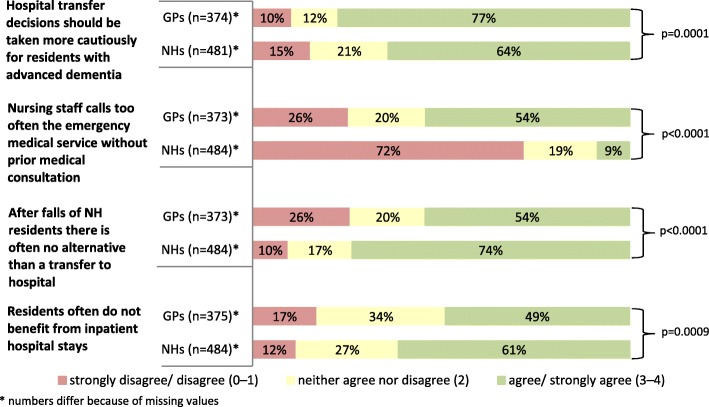
Responses to statements regarding hospital admissions and emergency department visits of nursing home residents – comparison between general practitioners (GPs) and nursing home staff (NHs)

Regarding current practices of hospital transfers most of the GPs and NH staff agreed on three of the four statements, albeit with different proportions of agreement (see Fig. 1). The bulk of all respondents thought that the decision for a hospital transfer should be taken more cautiously for NH residents with advanced dementia. The proportion of agreement was higher among the GPs (77.3%) than for NH staff (64.0%). More than half of the physicians (54.2%) shared the view that the nursing staff calls too often the emergency medical service without prior medical consultation while only 8.5% of the NH staff shared this opinion. Almost three quarters (73.6%) of the responding NH staff saw no alternative to a hospital transfer after a fall and 54.2% of the GPs had the same opinion. Many respondents agreed that NH residents often do not benefit from hospital admissions and the proportion was higher among NH staff than GPs (61.4% vs. 48.8%).

Of the measures for improvement we listed in the questionnaire, GPs and NH staff rated most differently (see Table 2), however, both groups saw the importance of almost all areas to reduce hospital transfers. Three measures with highest level of agreement among the GPs (each one: approx. 90%) were more nursing staff, better communication between nursing staff and GP, and qualification activities for nursing staff. The NH staff rated the importance of all of these lower with proportions around 60%. The three areas rated as most important by the NH staff were better GP’s care/availability (82.8%), better medical specialist’s care/availability (81.3%), and explicit ADs (72.4%). Notably, the second one was rated substantially less important by the GPs. A similar discrepancy could be found for the rating of better communication between nursing staff with much higher agreement among the GPs (80.0% vs. 35.7% in the NH survey). The presence of ADs was the only measure rated almost equally in its importance by also approx. Three quarters of GPs (*p* = 0.1492). In comparison with the other measures, the better availability of (medical) resources in the NH was assessed less relevant by both groups.

**Table 2:** Rating the importance of measures to reduce the number of hospital transfers – comparison between general practitioners (GPs) and nursing home staff (NHs)

Measure		none/ minor (0–1)	moderate (2)	major/ high (3–4)	p-value
More nursing staff	GPs (*n* = 370)*	2.4%	6.0%	91.6%	< 0.0001
	NHs (n = 476)*	22.3%	16.8%	60.9%	
Better communication between nursing staff and general practitioner	GPs (n = 373)*	3.0%	6.2%	90.9%	< 0.0001
	NHs (*n* = 479)*	15.5%	20.7%	63.9%	
Qualification activities for nursing staff	GPs (*n* = 372)*	1.6%	8.9%	89.5%	< 0.0001
	NHs (*n* = 481)*	19.8%	20.2%	60.1%	
Better communication between nursing staff	GPs (*n* = 369)*	7.1%	13.0%	80.0%	< 0.0001
	NHs (*n* = 468)*	40.1%	23.9%	35.7%	
Explicit advance directives	GPs (n = 372)*	11.3%	11.6%	77.2%	0.1492
	NHs (*n* = 482)*	11.4%	16.2%	72.4%	
Better general practitioner’s care/availability	GPs (n = 372)*	11.6%	25.5%	62.9%	< 0.0001
	NHs (*n* = 483)*	7.5%	9.7%	82.8%	
Better availability of (medical) resources in the nursing home	GPs (n = 371)*	21.0%	23.7%	55.3%	< 0.0001
	NHs (n = 481)*	36.2%	19.5%	44.3%	
Better medical specialist’s care/availability	GPs (n = 372)*	40.9%	23.4%	35.8%	< 0.0001
	NHs (*n* = 477)*	9.2%	9.4%	81.3%	

*numbers differ because of missing values

42 GP and 55 NH questionnaires also contained free-text responses in this section (complete data not shown). In both groups some of the responses stated here were repetitions of the measures already listed before, e.g., more nursing staff, better GP’s care/availability or qualification activities for nursing staff. Other mentioned aspects by the GPs included closer involvement of relatives or legal guardian/proxy (*n* = 10), expansion of responsibilities of nursing staff (*n* = 4) and creating a reliable jurisdictional basis for nursing procedures (*n* = 3). Most of the free-text responses made by NH staff referred to closer involvement of relatives or legal guardian/proxy (*n* = 18), followed by the clarification of jurisdictional questions (*n* = 6) and an improved end-of-life care in NHs (*n* = 5).

## Discussion

### Comparison of the findings with the existing literature

Based on two cross-sectional studies we found that GPs estimate the proportion of inappropriate hospital transfers higher than NH staff. On the contrary, more nursing staff agreed that residents do often not benefit from hospital admissions. Besides, GPs tended to the view that the nursing staff decides too soon in favour of a hospital transfer. Regarding areas for possible improvement, both groups rated very similarly the presence of explicit ADs. The importance of the NH-related measures was rated higher by the GPs while the nursing staff focussed on physicians’ care and availability.

The GP survey suggests that 35% of hospital admissions and almost 40% of ED visits among NH residents are inappropriate. The latter finding is in line with two other studies where physicians judged the inappropriateness of transferring NH residents to EDs with proportions of 33% [42] and up to 40% [40]. A structured implicit review of medical records investigating both types of hospital transfers identified 36% of ED visits and 40% of hospital admissions as not appropriate [35]. Remarkably, those findings also are in line with ours although that study was conducted in the US [35].

The NH staff estimated the proportions of both ED presentations and hospital admissions considerably lower than the GPs. This was also found in an US study by Ouslander et al. [38] when the involved nursing staff rated 23% of acute hospital transfers as potentially preventable. Vasilevskis et al. [55] compared the perspectives from hospital-based physicians and skilled nursing facility based staff on the avoidability of hospital readmissions of Medicare patients discharged to skilled nursing facilities. The authors found similarly that the nursing staff were less likely to rate these hospital visits as avoidable than the physicians. Further studies are needed to assess reasons for differences in ratings between various healthcare professionals.

Harrison et al. [39] used a series of vignettes based on common clinical scenarios and found that Scottish physicians and nurses most often agreed that the admission for the case with advanced dementia was inappropriate. This finding is comparable with ours. Over three quarters of GPs and almost two thirds of NH staff agreed that hospital transfer decisions should be taken more cautiously for those residents. However, a German study using claims data showed that hospitalisation rates of NH residents with dementia are almost as high as of those without dementia [56].

Interestingly, the proportions of agreement that NH residents often do not benefit from hospital admissions were in both surveyed groups higher than their assessed proportions of inappropriate hospital transfers. These findings seem to be conflicting at first sight. This especially occurs for the NH staff, since the nurses see the resident after discharge prior to the GPs in most cases and perceive the health status decline immediately. On the other hand, NH staff often considers no alternative than initiating a hospital transfer in our survey. This underlines that NH staff is often challenged by the complexity of hospital transfer decisions [37]. A multiplicity of factors influence the nurses to transfer a resident in a case of acute deterioration including family pressure [57], inability to provide a treatment on-site, and legal considerations [46, 58]. Taken these together, conflicts and uncertainties may arise making nurses more likely to decide in favour of a hospital transfer compared to physicians. An Austrian study explored that most of unplanned transfers are initiated by nurses without physician involvement [23]. This is also supported by our finding that the majority of GPs thought that the NH staff initiates transfers to hospital too often.

Thus, it is not surprising that from the GPs’ and the NH staff’s perspectives the importance of measures to reduce hospital transfers differs. Physicians put the emphasis on NH-related factors and rated most importantly the improvement of the staffing capacity in NHs. Physicians’ concerns about understaffing have also been identified in other studies in England [48] and France [54]. In the same way, the staffing level plays a key role in the facility staff decision-making [34, 46]. Further, an adequate training of the nurses is essential for a high quality of care [35] - about 90% of the GPs and 60% of the NH staff saw a need for action in this context. Diagnostic and treatment resources (e.g., oxygen, medications) available in the NH can be helpful [27, 33, 36, 46]. Inadequate skills in the assessment of first signs of deterioration can result in further decline [43]. However, additional time needed for such residents limits the staff’s availability to care for others increasing tendency for hospital transfers [59, 60]. Consequently, increasing the staffing ratio and continuous qualification activities are two key improvement measures [34–37, 46, 54, 59].

Early appropriate medical care and can be facilitated by improved GP’s availability during office hours and out-of-hours [33, 35, 36, 40, 46] and may also improve the patient-physician relationship. For the NH staff in our survey this was the most important measure directly followed by the demand for better medical specialist’s care and availability. Predominantly, the GPs agreed to the first point; however, they disagreed to improve specialist’s care. GP’s coordination function is estimated to be even more important for NH residents since GPs tend to have a greater expertise in the care of this frail population. Specialists’ contacts or treatment decisions without GP’s involvement contrast with this role. On the other side, the NH staff might think that GPs have less expertise in providing adequate care in all possible scenarios - although in Germany, GPs provide the bulk of medical care in this population [4, 44]. Such disagreements can be caused by communication difficulties between nurses and physicians and uncertain responsibilities which are known to contribute to acute hospital transfers of NH residents [46]. Sharing information about a resident’s condition between nurses [33, 34, 54] and between nursing staff and GP [33, 34, 37, 41, 46, 48] has the potential to prevent inappropriate hospital transfers. This is supported by our surveys in which both groups rated the interprofessional communication highly important. For instance, Dutch NHs employ next to the nursing staff specialized elderly care physicians (formerly NH physicians) [61, 62] who provide a continuity of care which can reduce potentially inappropriate hospital transfers [63]. Concerning the communication among the staff we could see a larger discrepancy. Whereas the GPs rated this measure important, which was also shown in two studies in France [54] and the UK [34], the majority of the responding NHs perceived no problem here.

For both GPs and nurses, the availability of an AD can be a support to make hospital transfer decisions in better accordance with the resident’s wishes [27, 33, 35, 42]. In our two surveys, it was the only measure for improvement rated in its importance very similar by both groups (approx. 75% agreement, resp.). Nevertheless, only a minority of residents is estimated to have a personal AD [64] and problems in their use such as the often insufficient specificity are known [40, 64, 65]. Advance care planning (ACP) aims to discuss and record patient preferences concerning goals of care in the case of physical or mental deterioration [66] and a German study showed that its implementation leads to a better adoption of ADs in NHs [67]. A randomized controlled trial on the implementation of an AD program in Canadian NHs [68] indicates less hospital admissions in residents with ADs [68]. Thus, increasing the prevalence and the validity of ADs by further implementation of ACP could facilitate medical decision-making and prevent hospital transfers. This process should ideally start before the NH placement.

### Limitations and strengths

Some of the findings of this study, especially the stated proportions of inappropriate hospital transfers have to be interpreted with caution as they are attributed to personal impressions of the responding GPs and NH staff. There exists a broad range of ways to define appropriateness of transfers [24, 25] but this study aimed to illustrate the perception of GPs and NH staff in Germany. Another limitation applies to the generalizability of the findings. On the one hand, we could include facilities from all over Germany in our NH sample; however, we had only access to GP data from two federal states’ Associations of the Statutory Health Insurance Physicians. However, a comparison of all NHs’ answers with the ones from Bremen and Lower Saxony showed only slight differences. At the same time, we were nearly able to achieve the target response in the GP survey (33.5%). In the NH survey we even attained a higher response than expected (45.5%). By using several strategies shown to increase the response of postal surveys [52] our GPs’ response ranges within other questionings among GPs in Germany [51, 69]. In the NH survey we even had a higher proportion of returned questionnaires compared to other recently published studies conducted with German NHs [70, 71]. However, for both surveys a selection bias cannot be ruled out. The presented *p*-values were calculated in exploratory analyses and they were not adjusted for multiple testing since sample size calculation was not originally planned for the comparison of GPs and NH staff. Although we showed in this study the views of two important groups there are of course other perspectives which should be obtained in future studies (from paramedics, hospital physicians, transferred residents and their family members).

## Conclusions

GPs and NH staff are two main groups for assessing the appropriateness of transfers from NH to hospital. Although German NH residents are often transferred research about the perspectives of these healthcare professionals from Germany is scarce. In these two cross-sectional studies we tried to close this gap and found both comparable and also to some extent different perspectives of GPs and NH staff on acute hospital transfers. Although nurses considered to a lesser degree transfers inappropriate both groups thought that too many potential burdensome hospital admissions and ED visits occur. Our findings support the international evidence that improving the interprofessional communication and collaboration is essential to reduce the number of hospital transfers. Higher staffing levels and an improved education with a better availability of GPs can empower the nurses managing acute situations more confident and adequately. Besides, knowing and respecting the resident’s perspective and autonomy is another important issue avoiding inappropriate hospital transfers. As our study showed the high complexity of hospital transfer decisions in this population, future research on planning and evaluating interventions for reducing inappropriate transfers to hospitals should take this broad range of aspects and professionals into account.

## Supplementary information

**Additional file 1:** Original version of the questionnaire on general practitioners.

## Data Availability

The datasets supporting the conclusions of this article are available from the corresponding author on reasonable request.

## Abbreviations

ACP: advance care planning
ACSC: ambulatory care sensitive condition
AD: advance directive
CI: confidence interval
ED: emergency department
GP: general practitioner
NH: nursing home
SD: standard deviation
UK: United Kingdom
US: United States
