# Supplementary material for: Perspectives of general practitioners and nursing staff on acute hospital transfers of nursing home residents in Germany: results of two cross-sectional studies
Source: BMC Fam Pract. 2020 Feb 11;21:29. doi: 10.1186/s12875-020-01108-x (PMC7014634; doi:10.1186/s12875-020-01108-x)
Supplement: Supplementary file 1 — Additional file 1: Original version of the questionnaire on general practitioners. [file 12875_2020_1108_MOESM1_ESM.pdf]

Einschätzung von Hausärzten zur Versorgung von Pflegeheimbewohnern

HOMERN

Bei Rückfragen wenden Sie sich bitte an:

Carl von Ossietzky universität Oldenburg | Universität Bremen

Ove Spreckelsen | Carl von Ossietzky Universität Oldenburg,
Department für Versorgungsforschung, Abteilung Allgemeinmedizin
Telefonnr.: 0441 798-4408 | E-Mail: HOMERN@uni-oldenburg.de

Im folgenden Fragebogen geht es um die Beurteilung der medizinischen Versorgung von Heimbewohnern* aus hausärztlicher Sicht. Der hier verwendete Begriff „Krankenhaustransport“ umfasst sowohl stationäre Aufenthalte als auch ambulante Notaufnahmebesuche.

*Zu Gunsten der besseren Lesbarkeit wird im Fragebogen ausschließlich die männliche Form verwendet (z.B. Heimbewohner, Hausärzte). Frauen sind dabei selbstverständlich in gleicher Weise angesprochen.

Questions used for this study: 4,5,6,11,12

Medizinische Versorgung in Pflegeheimen

1

a) **Stimmen Sie der Aussage zu, dass die Versorgung von Heimbewohnern vom Hausarzt koordiniert werden sollte?**

☐ nein ☐ ja

b) **Sollte die Behandlung von Heimbewohnern durch andere Fachärzte in der Regel nur nach Überweisung durch Hausärzte erfolgen?**

☐ nein ☐ ja

2

Wie schätzen Sie den Versorgungsbedarf durch folgende Arztgruppen ein?

	sehr gering 0	1	2	3	sehr hoch 4
Augenärzte	☐	☐	☐	☐	☐
Chirurgen/ Orthopäden	☐	☐	☐	☐	☐
Dermatologen	☐	☐	☐	☐	☐
Gynäkologen	☐	☐	☐	☐	☐
Hals-Nasen-Ohrenärzte	☐	☐	☐	☐	☐
Psychiater/ Neurologen	☐	☐	☐	☐	☐
Urologen	☐	☐	☐	☐	☐
Zahnärzte	☐	☐	☐	☐	☐

3

Inwieweit stimmen Sie den folgenden Aussagen zu?

	gar nicht 0	1	2	3	voll 4
Die Weiterbildung stellt eine ausreichende Qualifikation für die hausärztliche Versorgung von Heimbewohnern dar	☐	☐	☐	☐	☐
Die Versorgung aller Bewohner eines Heimes nur durch eine Praxis führt (im Vergleich zu mehreren Praxen) zu einer höheren Qualität der Versorgung	☐	☐	☐	☐	☐
Speziell qualifizierte Medizinische Fachangestellte (MFA), die regelmäßig Heimbewohner besuchen, sind eine wichtige Unterstützung	☐	☐	☐	☐	☐
Bei der Versorgung von Heimbewohnern ist der Kassenärztliche Bereitschaftsdienst eine wichtige Unterstützung	☐	☐	☐	☐	☐
Für die medizinische Versorgung in Heimen können telemedizinische Konsile hilfreich sein	☐	☐	☐	☐	☐

Versorgung im Krankenhaus und der Notaufnahme

4 **Insgesamt betrachtet, welchen Anteil stationärer Krankenhausaufenthalte und ambulanter Notaufnahmebesuche von Heimbewohnern schätzen Sie als nicht erforderlich ein?**

........% der Krankenhausaufenthalte ....% der Notaufnahmebesuche

5 **Inwieweit stimmen Sie den folgenden Aussagen zu?**

	gar nicht 0	1	2	3	voll 4
Von stationären Krankenhausaufenthalten profitieren Heimbewohner oftmals nicht	☐	☐	☐	☐	☐
Pflegekräfte rufen zu häufig den Rettungsdienst ohne vorherige ärztliche Rücksprache	☐	☐	☐	☐	☐
Bei Stürzen von Heimbewohnern gibt es oft keine Alternative zu einem Transport ins Krankenhaus	☐	☐	☐	☐	☐
Bei Heimbewohnern mit fortgeschrittener Demenz sollte die Indikation für einen Krankenhaustransport im Allgemeinen zurückhaltender gestellt werden	☐	☐	☐	☐	☐

6 **Welche Bedeutung haben aus Ihrer Sicht die folgenden Maßnahmen, um Krankenhaustransporte von Heimbewohnern zu reduzieren?**

	keine 0	1	2	3	große 4
bessere Kommunikation zwischen Pflegekräften	☐	☐	☐	☐	☐
bessere Kommunikation zwischen Pflegekräften und Hausarzt	☐	☐	☐	☐	☐
bessere Versorgung/ Erreichbarkeit durch Hausarzt	☐	☐	☐	☐	☐
bessere Versorgung/ Erreichbarkeit durch Facharzt	☐	☐	☐	☐	☐
bessere Ausstattung des Heims (z.B. Katheter, Schnelltests, Medikamente)	☐	☐	☐	☐	☐
bessere Personalausstattung der Heime	☐	☐	☐	☐	☐
Qualifizierungsmaßnahmen für das Pflegepersonal	☐	☐	☐	☐	☐
aussagekräftige Patientenverfügung	☐	☐	☐	☐	☐
Sonstiges:	☐	☐	☐	☐	☐

Versorgung am Lebensende

7 **Bitte schätzen Sie die Situation in Deutschland ein:**

a) Wie hoch ist der Anteil an Heimbewohnern mit einer Patientenverfügung?

........%

b) Wie häufig sind diese Verfügungen aussagekräftig in Bezug auf Krankenhaustransporte am Lebensende?

........%

c) Wie häufig werden diese Verfügungen nicht dem Bewohnerwunsch entsprechend berücksichtigt?

........%

Questions used for this study: 4,5,6,11,12

8 Inwieweit stimmen Sie den folgenden Aussagen zu?

	gar nicht 0	1	2	3	voll 4
Heimbewohner werden am Lebensende zu häufig im Krankenhaus behandelt	☐	☐	☐	☐	☐
Heimbewohner müssten häufiger in die spezialisierte ambulante palliativmedizinische Versorgung (SAPV) eingeschrieben werden	☐	☐	☐	☐	☐
Pflegepersonal der Heime sollte besser für die Versorgung am Lebensende ausgebildet werden	☐	☐	☐	☐	☐
Hausärzte sollten für die Versorgung am Lebensende auch außerhalb der Sprechzeiten erreichbar sein	☐	☐	☐	☐	☐
Hausärzte sind in der Regel gut für die Versorgung von Heimbewohnern am Lebensende ausgebildet	☐	☐	☐	☐	☐

9 Bitte schätzen Sie die Situation in Deutschland ein:

a) Wie hoch ist der Anteil an Heimbewohnern, der im Krankenhaus und nicht im Heim verstirbt?

.... % der verstorbenen Heimbewohner

b) Wie hat sich der Anteil im Krankenhaus verstorbener Heimbewohner in den letzten 10 Jahren verändert?

☐ ist zurückgegangen ☐ blieb unverändert ☐ ist angestiegen

c) Wie ist die Versorgung von Heimbewohnern am Lebensende insgesamt?

☐ eher schlecht ☐ eher gut

→ **d) Falls Sie mit „eher schlecht" geantwortet haben, was wäre nach Ihrer Einschätzung die wichtigste Maßnahme, mit der die Versorgung verbessert werden könnte?**

....

....

Versorgung eines individuellen Falls

10 Bitte lesen Sie sich folgendes Szenario durch:

Es ist Samstagabend gegen 22:00 Uhr. Die Pflegekraft misst bei einer wachen und ansprechbaren 86-jährigen Heimbewohnerin eine Temperatur von 38,1°C, die Atemfrequenz ist 14/min, RR 140/80mmHg, Puls 85/min, periphere Sauerstoffsättigung 96%. Die Bewohnerin klagt bereits seit drei Tagen über unproduktiven Husten, Abgeschlagenheit und körperliche Schwäche. Die Heimbewohnerin ist leicht dement. Die Pflegekraft alarmiert den Rettungsdienst, der die Patientin ins Krankenhaus transportiert.

a) Wie beurteilen Sie in diesem Fall die Entscheidung zum Krankenhaustransport?

☐ eher nicht gerechtfertigt ☐ eher gerechtfertigt

b) Was ist für Sie der maßgebliche Grund, diesen Transport als gerechtfertigt bzw. nicht gerechtfertigt einzuschätzen?

....

....

Questions used for this study: 4,5,6,11,12

10 c) Wie beurteilen Sie in diesem Fall die Entscheidung zum Krankenhaustransport, wenn…

	eher nicht gerechtfertigt	eher gerechtfertigt
...die Patientin dauerhaft bettlägerig gewesen wäre	☐	☐
...der nächste Angehörige sich in der Vergangenheit gegen unnötige Krankenhaustransporte ausgesprochen hätte	☐	☐
...sich der Zustand der Patientin seit dem letzten Besuch des Hausarztes vor zwei Tagen nicht verändert hätte	☐	☐
...eine Infusionstherapie im Heim durch das Pflegepersonal möglich wäre	☐	☐
...es eine Möglichkeit der notfallmäßigen Kontaktaufnahme zum behandelnden Hausarzt gegeben hätte	☐	☐

Angaben zu Ihrer Person

11 a) Alter: ....Jahre

b) Geschlecht: ☐ männlich ☐ weiblich

c) Wie lange sind Sie hausärztlich als Facharzt/ praktischer Arzt tätig?

............Jahre

d) In welcher Art von Praxis arbeiten Sie?

☐ Einzelpraxis ☐ Gemeinschaftspraxis/ Praxisgemeinschaft ☐ Medizinisches Versorgungszentrum (MVZ)

e) Verfügen Sie über eine oder mehrere der folgenden Zusatzbezeichnungen?

☐ Palliativmedizin ☐ Geriatrie ☐ Notfallmedizin

f) Wie oft haben Sie im letzten Jahr am KV-Bereitschaftsdienst teilgenommen?

............mal

g) Wie groß ist der Ort, in dem Ihre Praxis liegt?

☐ bis 2.000 Einwohner ☐ bis 5.000 Einwohner ☐ bis 20.000 Einwohner
☐ bis 50.000 Einwohner ☐ bis 100.000 Einwohner ☐ mehr als 100.000 Einwohner

Ihre Erfahrungen mit der Versorgung in Pflegeheimen

12 a) In wie vielen Heimen versorgen Sie Bewohner?

............Heime

b) Wie viele Heimbewohner versorgen Sie insgesamt?

ca. ....Heimbewohner

c) Wie häufig sind Sie im letzten Quartal in dem Heim gewesen, in dem Sie die meisten Bewohner versorgen?

ca. ....mal

d) Sind Sie oder Praxiskollegen in dringenden Fällen außerhalb der Praxisöffnungszeiten für das Pflegepersonal telefonisch erreichbar?

☐ nein ☐ ja

e) Welchen Anteil der Heimbewohner, die im Krankenhaus stationär behandelt wurden, sehen Sie innerhalb der ersten Woche nach Entlassung?

☐ 0-25% ☐ 26-50% ☐ 51-75% ☐ 76-100%

Vielen Dank für Ihre Teilnahme!

Questions used for this study: 4,5,6,11,12
